# An In-Ear PPG-Based Blood Glucose Monitor: A Proof-of-Concept Study

**DOI:** 10.3390/s23063319

**Published:** 2023-03-21

**Authors:** Ghena Hammour, Danilo P. Mandic

**Affiliations:** Department of Electrical and Electronic Engineering, Imperial College London, London SW7 2AZ, UK

**Keywords:** blood glucose, continuous monitoring, diabetes, hearables, in-ear PPG, machine learning, non-invasive, photoplethysmography (PPG), NIR spectroscopy

## Abstract

Monitoring diabetes saves lives. To this end, we introduce a novel, unobtrusive, and readily deployable in-ear device for the continuous and non-invasive measurement of blood glucose levels (BGLs). The device is equipped with a low-cost commercially available pulse oximeter whose infrared wavelength (880 nm) is used for the acquisition of photoplethysmography (PPG). For rigor, we considered a full range of diabetic conditions (non-diabetic, pre-diabetic, type I diabetic, and type II diabetic). Recordings spanned nine different days, starting in the morning while fasting, up to a minimum of a two-hour period after eating a carbohydrate-rich breakfast. The BGLs from PPG were estimated using a suite of regression-based machine learning models, which were trained on characteristic features of PPG cycles pertaining to high and low BGLs. The analysis shows that, as desired, an average of 82% of the BGLs estimated from PPG lie in region A of the Clarke error grid (CEG) plot, with 100% of the estimated BGLs in the clinically acceptable CEG regions A and B. These results demonstrate the potential of the ear canal as a site for non-invasive blood glucose monitoring.

## 1. Introduction

Diabetes is a chronic illness that affects 537 million adults around the world and this number is expected to rise to 783 million people by 2045 [1]. People suffering from diabetes experience abnormal blood glucose levels (BGL), mainly owing to the absence or unregulated production of insulin. For example, in the case of type II diabetes, which accounts for 90% of the cases, the body does not produce enough insulin or is resistant to it, hence causing prolonged high levels of blood glucose [2]. When left unmanaged and untreated, diabetes causes serious health problems and complications that can affect the eyes, heart, kidneys, and blood vessels. Diabetes may even have life-threatening consequences [3], with 6.7 million people worldwide losing their lives to diabetes in 2021 [4]. However, this chronic illness can be cured or delayed when it is in its pre-diabetic stage, and its complications prevented under the correct diet, improved lifestyle, and constant BGL monitoring, a subject of this study.

To minimize the complications and adverse health effects of diabetes, it is crucial for people with diabetes (PWD) to monitor their BGLs several times per day (4–10 times), typically before and after meals, and especially if they are under insulin treatment [5]. Currently available out-of-clinic blood glucose monitoring (BGM) devices include invasive and minimally invasive ones. Invasive BGM involves pricking the finger to extract a small amount of capillary blood using a lancet (small needle). The blood is then collected on a strip which is inserted into a portable machine that measures and displays the BG level [6]. This method is not only painful, but also carries the risk of infection, and does not provide a continuous means for BGM. In addition, it is unsuitable for a wide range of populations, the elderly for example, who might lack fine control of their hands and fingers or have poor eyesight which prevents them from performing all the required steps of the measurement [7].

Minimally invasive devices, on the other hand, do provide a more continuous form of BGM. However, these devices typically involve the temporary implantation of a micro-needle that senses BGL from the interstitial fluid. The main problems with such devices include drifts and lags between the BGL from the interstitial fluid versus true BGL, in addition to the high running cost associated with an implanted needle that needs to be disposed of every few days [8].

With the ever-growing number of PWD and the shortcomings of current BGM systems, there is an urgent need for readily wearable, non-invasive devices for continuous BGM. In recent years, several such non-invasive methods have been investigated; these either rest upon the sampling of bodily fluids, such as urine, tears, and sweat, or are based on shining a light of a certain wavelength through the skin [9]. While each method has its advantages and disadvantages [10], optical methods, especially those pertaining to near-infrared (NIR) spectroscopy, has attracted much interest. The NIR region of the electromagnetic spectrum lies between 700–2500 nm. It penetrates the skin in the mm range, depending on the wavelength [11], and has been adopted for the development of continuous non-invasive BGM devices at various body locations [12,13,14,15,16,17,18]. These devices mainly derive the glucose level from features extracted from photoplethysmography (PPG) waveforms, which can be acquired from any part of the skin that has blood vessels [19]—most commonly from the fingers, wrist, and earlobes. The PPG sensor used typically comprises light-emitting diodes (LED) of a certain wavelength(s) and a photodiode that receives the reflected light and is used to measure changes in blood volume in the tissue. It typically involves shining a light onto the skin and measuring the light intensity that is reflected back. This measurement is then used to calculate the amount of blood in the tissue and can provide information about heart rate, blood pressure, and other physiological parameters.

Recent advancements in wearable technology for vital signs monitoring have given rise to in-ear wearable devices, the so-called hearables [20,21]. Given the privileged position of the head on the human body, the ear provides a convenient and stable site for physiological measurement, while the ear canal also acts as an insulator of external electrical noise [21]. Several studies have used the ear canal for general physiological sensing which includes electroencephalography (EEG) [22,23,24], electrocardiography (ECG) [25], and PPG [26,27,28], and have reported promising results when compared to clinical devices.

This work introduces the proof of concept for a continuously wearable in-ear non-invasive glucose monitoring system that uses a single NIR LED (880 nm) to acquire the PPG signal. For rigor, we considered a whole range of diabetic conditions—a non-diabetic control, pre-diabetic, type I, and type II diabetic subject with the considered BGL range varying from 63–345 mg/dL. The blood glucose levels were estimated based on 18 discriminating features, which were employed by machine learning algorithms to predict the BGL values from the corresponding PPG waveforms. The input variable selection (IVS) across all subjects indicates that the most discriminative features are related to the energy of the PPG cycle. As desired, all of the predicted BGLs fell in regions A and B of the Clarke error (CEG) plots—the regions with no adverse effects on clinical outcomes. To the best of our knowledge, the proposed system is the first to utilize the ear canal as a site of non-invasive blood glucose measurement through NIR spectroscopy and opens up avenues for unobtrusive 24/7 blood glucose monitoring in the community.

## 2. Materials and Experimental Design

### 2.1. Working Principle

**Figure 1:**
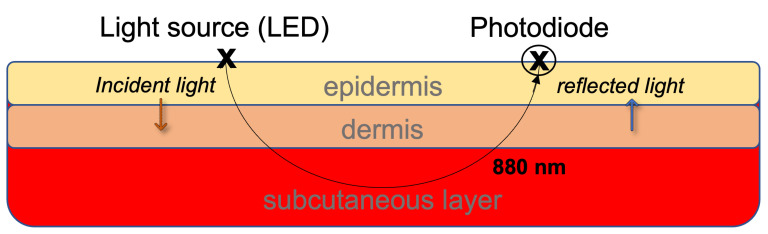
The principle of light reflectance and absorption through the different layers of the skin.

When the NIR light is emitted through the blood vessels in the ear canal, it interacts with the medium and is reflected, as shown in Figure 1. Its intensity is reduced due to both absorption and scattering [17] so that pulsed-periodic signals are recorded, known as a photoplethysmograph (PPG). The principle of PPG is that blood with a higher concentration of glucose will have different absorbance than blood with lower glucose levels. This will affect the shape of the PPG signal which can then be used to indirectly estimate the blood glucose level through a mapping onto a set of features that are fed into the machine learning algorithms [12,13,14,15,17,18,29].

### 2.2. Hardware Design

**Figure 2:**
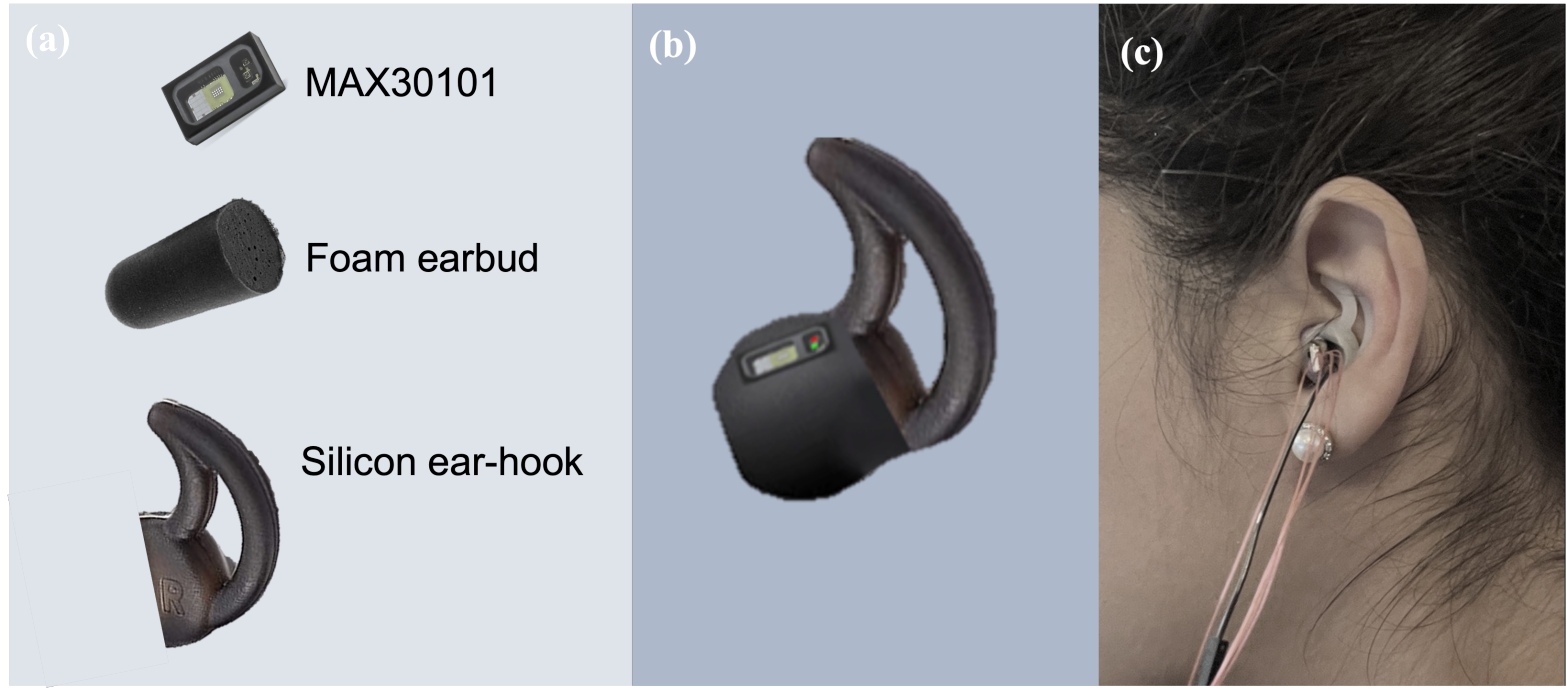
The in-ear PPG sensor used in our study. (**a**) The different components of the in-ear sensor. (**b**) The PPG sensor chip embedded on a viscoelastic earbud attached to an ear hook. (**c**) Sensor placement within the ear canal.

In this study, we used the infrared (880 nm) LED of the MAX30101 digital PPG chip by Maxim Integrated (San Jose, CA, USA), which has been used in a number of studies based on in-ear PPG acquisition [26,27,28]. The PPG chip was positioned on a thin rectangular printed circuit board with decoupling capacitors and was then embedded onto a viscoelastic foam earpiece, and attached to a silicon-based ear hook as shown in Figure 2. For user comfort and sensor stability, the ear hook was available in three different sizes; small, medium, and large. The output of the PPG chip was then connected to a data acquisition board with a Bluetooth-enabled microcontroller (Nordic nRF52840), Nordic Semiconductor, Trondheim, Norway which transferred the data in real time to a PC where it was stored and processed. The collection of reference values of BGLs was performed by a commercially available glucometer (Accu-Check Performa Nano), whose accuracy meets the DIN EN ISO 15197:2013 requirements of a measuring interval; that is, 10–600 mg/dL.

### 2.3. Experimental Design

**Figure 3:**
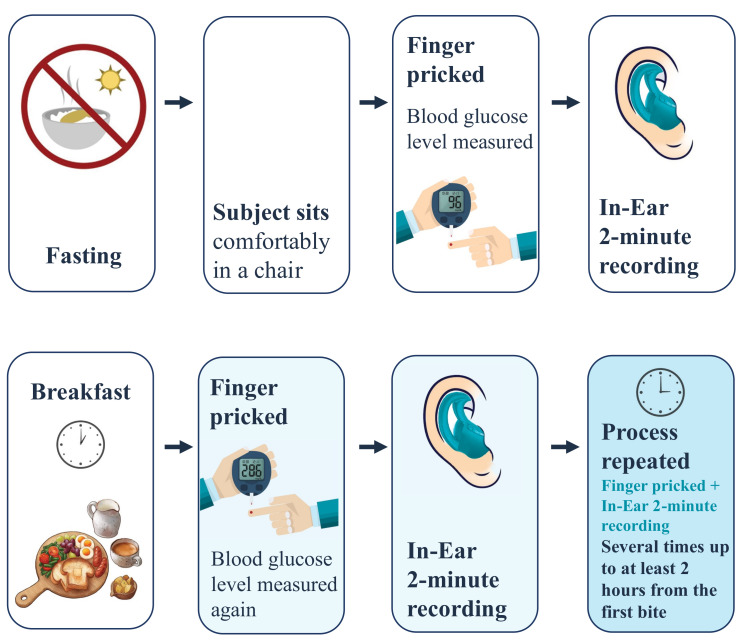
The recording protocol in this study.

Four subjects, aged between 30 and 59 years, were recruited for this study, with one non-diabetic female, one pre-diabetic male, one type I diabetic male, and one type II diabetic male (Table 1). The participants were given detailed instructions and explanations about the recording protocol and were asked to attend the recording sessions in the morning while still fasting. Each participant attended the recordings over the course of 9 days. The BGLs were measured using the gold standard glucometer, while simultaneously the in-ear PPG signal was recorded for two minutes (starting within one minute of the glucometer measurement), and each recording was repeated twice. The BGL and PPG were first measured when a subject was fasting, then a varied and carbohydrate-rich (where permissible) breakfast was offered. Afterward, the BGL and PPG signals were measured at various intervals and at least up to 2 h after breakfast, as depicted in Figure 3. The total number of two-minute recordings acquired was 244, and the glucose levels varied between 65 and 345 mg/dL. It is important to note that during each recording day, the subject was asked to keep the sensor in their ear to minimize the variability between recordings and examine the comfort and ergonomic aspects of the proposed device. In addition, the lancets and test strips were safely disposed of after every finger prick and the subjects were asked to wash their hands with lukewarm water before and after each recording. All subjects gave a written informed consent for inclusion before they participated in the study. The recordings were performed under Imperial College’s ethics committee approval JRCO 20IC6414.

## 3. Signal Processing/Methods

### 3.1. Pre-Processing of PPG Waveforms

**Figure 4:**
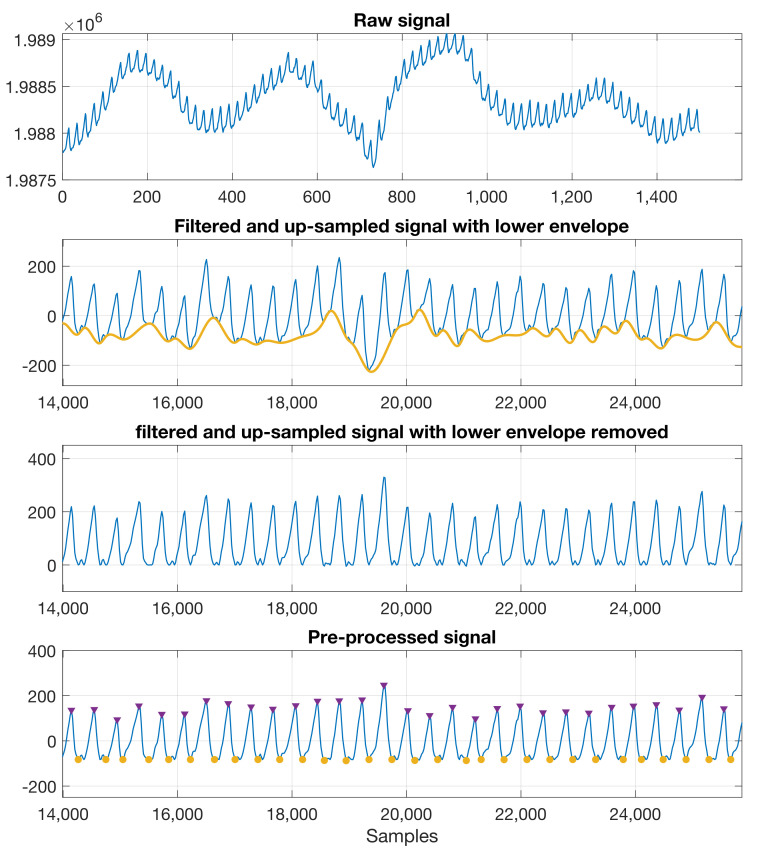
Pre–processing steps for the raw PPG. Top to bottom: raw signal; band–pass signal with the lower envelope detected; upsampled signal with the lower envelope removed; pre–processed signal with the peaks and troughs identified.

The recorded raw PPG signals were filtered through a 10th-order Butterworth low-pass filter with a cut-off frequency of 0.01 Hz, to acquire the DC component of PPG. For the AC component of the PPG signal, the raw data were filtered through an 8th-order Butterworth band-pass filter between 0.3 and 10 Hz. The signals were upsampled using linear interpolation to better locate the peaks and troughs. The lower envelope of the filtered signal was then detected using spline interpolation between the local minima and was subtracted to remove the baseline wander, so as to enable the identification of the peaks and troughs. Figure 4 shows a sample recording with the pre-processing steps.

### 3.2. PPG Cycle Analysis and Feature Extraction

**Figure 5:**
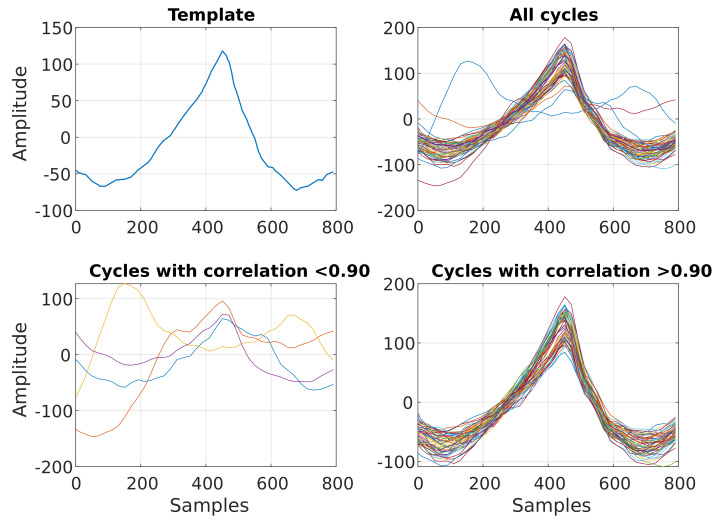
Matched filtering of PPG cycles. (**Top left**) Exemplar template used for matched filtering of PPG cycles. (**Top right**) Overlay plot of all PPG cycles acquired during one recording. (**Bottom left**) Exemplar low–quality PPG cycles, with a correlation < 0.9 with the template. (**Bottom right**) Overlay plot of the remaining PPG cycles after matched filtering.

In order to understand the variation of the PPG signal with the glucose level in order to extract features that best capture this relationship, we performed an analysis of the PPG cycles across the glucose levels. The PPG signal was divided into windows centered around the peak of each cycle, with each window containing a single cycle. The PPG cycles were selected for further analysis using a matched filter—a template cycle created by taking the mean of the middle ten cycles of each recording. After each PPG cycle was correlated with the template cycle, the cycles with a correlation value of r < 0.90 were deemed to be too noisy and removed from the analysis, as shown in Figure 5. In this way, 10–20% of the cycles were removed from recordings for each subject. The remaining valid cycles were then divided into those pertaining to low- and high-glucose cycles. For each subject, the average glucose level measured during fasting was used as a threshold to determine periods of low and high glucose levels. Low glucose cycles then correspond to the glucose levels below the threshold and high glucose cycles represent the glucose levels above a subject’s threshold.

**Figure 6:**
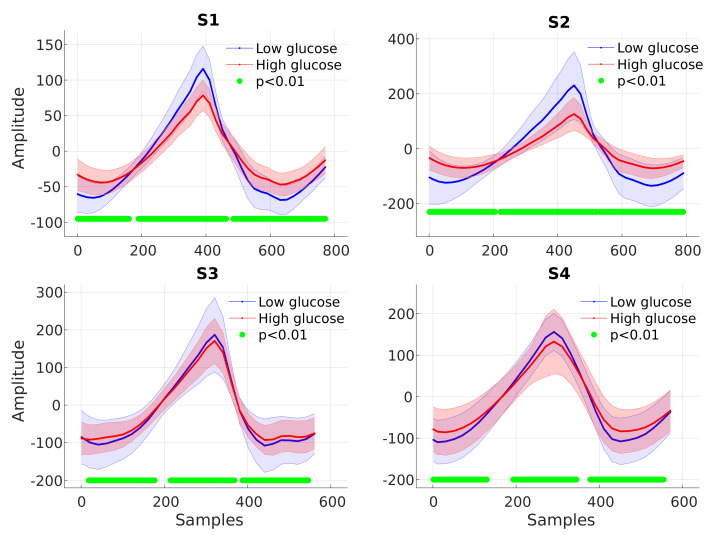
Averaged low–glucose (blue) and high–glucose (red) PPG cycles with the corresponding shaded areas indicating the confidence intervals. The green dots designate time instances where the null hypothesis of no statistical difference between the low–glucose and high–glucose PPG cycles is rejected.

**Figure 7:**
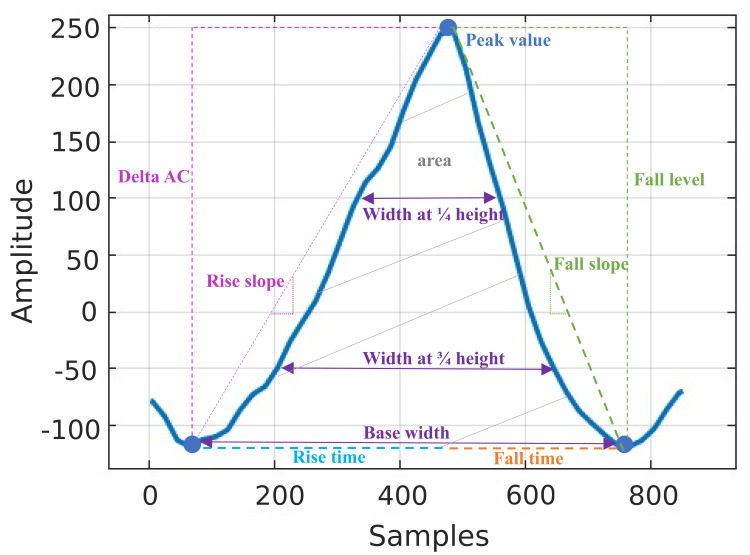
Features extracted from every PPG cycle.

The ensemble average PPG cycle was computed from high and low glucose cycles and compared between the two groups. Significant differences in the PPG cycle between the low and high glucose groups were detected by applying a two-sample *t*-test at each point in the cycle. Figure 6 shows that for all the subjects, the PPG cycles were found to have higher amplitudes and narrower peaks during low glucose periods. Based on this observation and the available literature [12,13,14,15,16,17,18,29,30,31], we created features that capture this change in the dynamics at different glucose levels, as shown in Figure 7. In addition to these features, the energy profile of each cycle was calculated using the Teager–Kaiser energy operator (TKEO), a feature commonly used to quantify the instantaneous energy profile in periodic components of signals [15,16,17,18]. Table 2 summarises all features extracted from every valid PPG cycle.

### 3.3. Glucose Prediction

Based on the set of features in Table 2, a suite of machine learning models was trained on each subject individually. Subject-specific training was necessary for this pilot study because each subject belongs to a different BG condition category. This was achieved using the first seven days as the training data (with a five-fold cross-validation) and the remaining two days as unseen test data. The models employed include linear regression, support vector machines (SVM), Gaussian process regression, kernel approximation models, ensemble models, and neural networks (NNs). The models were trained in the MATLAB programming environment, using machine learning and deep learning toolboxes [32]. The ensemble regression trees model resulted in the lowest root mean squared error (RMSE) value and was chosen for the analysis of the unseen data. To test the clinical significance of the test data, the Clarke error grid (CEG) was used [33], along with the mean absolute relative difference (MARD), the correlation coefficient r, and the RMSE.

## 4. Results

**Figure 8:**
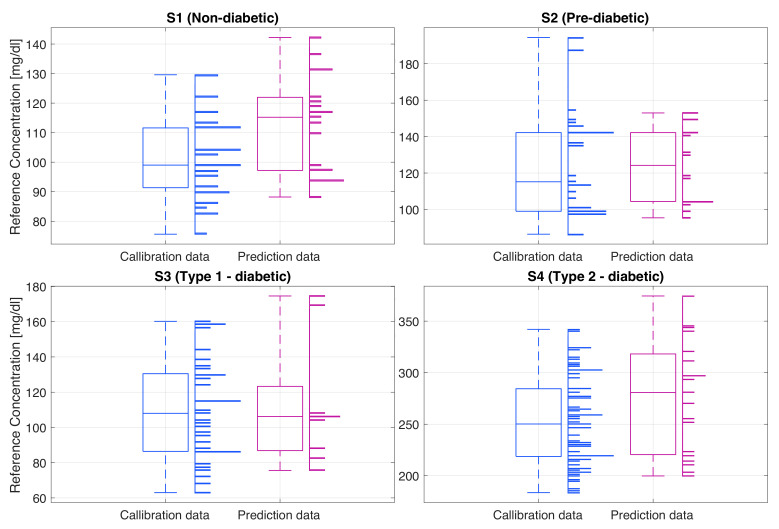
The distribution of the true glucose levels of the calibration and prediction data for each subject (S1–S4), recorded using the gold standard glucometer. Histograms (horizontal bars) show the relative number of measurements at different glucose levels (*y*-axis) and the box plots indicate the summary statistics of all recordings for each subject.

The gold standard glucose levels of the recorded subjects, shown in Figure 8, were consistent with their health conditions. For the non-diabetic subject (S1) the glucose ranges were within the normal limits, typically 90 mg/dL while fasting, and with a maximum of 142 mg/dL typically half an hour after the meal, after which the BGL dropped back to around 90 mg/dL. Subject 2 (S2), a pre-diabetic, had a higher fasting glucose level of around 100 mg/dL and a maximum glucose value of 194 mg/dL. High BGL remained constant over a longer period of time, typically returning to the fasting level within about two hours from breakfast. The type I diabetic subject (S3) had hypoglycemic BGLs. This subject was using insulin to regulate the BGL, typically right before the meal, after an estimate of the number of carbohydrates in food. For this type of diabetes, as evident from the data, the glucose levels tend to be quite unpredictable; for example, they can be low both before and after the mean, or equally, they can be high both before and after the meal. The highest BGL can be seen in the type II diabetic subject (S4) where the fasting glucose was around 200 mg/dL and the maximum BGL after the meal was around 354 mg/dL. It is important to note that this subject does not inject any insulin, hence the constantly elevated BGL.

**Figure 9:**
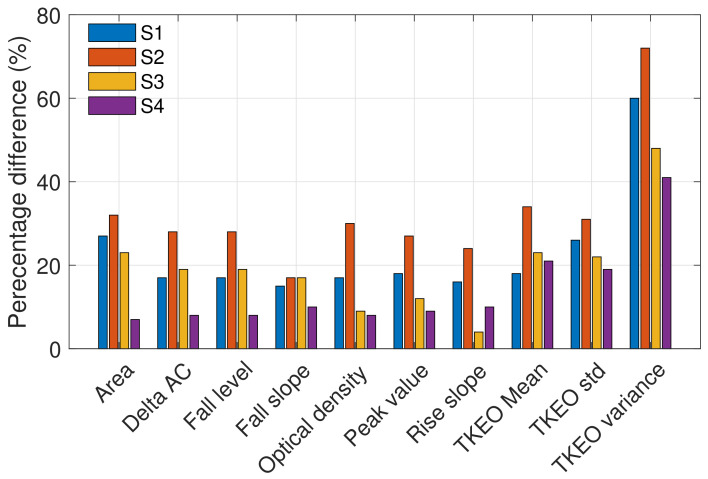
Percentage difference between the high and low glucose levels, based on the values of the ten most significant features across the four subjects. The % difference indicates the importance of a feature.

The PPG signals obtained from each subject using the in-ear PPG sensor showed clear systolic peaks, with an average of 80% of the cycles (no. of valid cycles in Table 3) having a high correlation (r > 0.9) with the template cycle of the recording in hand, as shown in Figure 5. The highest number of discarded cycles was for subject S3; this is likely due to the subject moving their mouth, grinding their teeth, yawning, coughing, or due to sensor displacement. The clean cycles were split between the training and test sets, with the first seven days used to train the model and the last two days for testing. The ten most significant features which yielded statistically significant differences in high versus low blood glucose PPG cycles are presented in Figure 9. Observe that the highest percentage differences are found for S1 and S2, while the lowest percentage difference can be observed in S4. Features calculated from the energy feature TKEO were constantly highly representative of BGLs in all subjects. The features extracted from training data were used to train different machine learning regression models. The best-performing models were ensemble regression trees; these models were optimized for each subject with hyperparameter ranges and validation results, as shown in Table 4.

**Figure 10:**
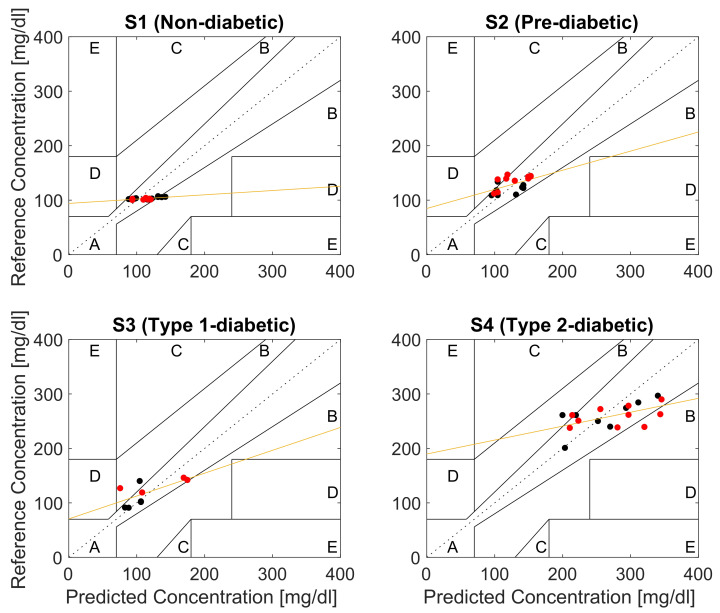
Clarke error grid analysis (EGA) of the glucose predictions for a non-diabetic subject S1, pre-diabetic subject S2, type I diabetic subject S3, and type II diabetic subject S4 (black dots: Day 8 samples, red dots: Day 9 samples, orange line: linear least-squares regression of the reference versus the predicted glucose concentrations).

Figure 10 shows the CEG plot for each subject. Observe that, as desired, all the predicted BGL values reside within regions A and B of the CEG plots thus indicating no adverse effect on clinical decisions based on the proposed device. Moreover, on average 80% of the data for all subjects lie within region A of the CEG. The best prediction results, with around 90% of the predicted points in region A, were obtained for S1, followed by 83.3% for S2, while the lowest predicted points of 77% in region A were for S3 and S4, as shown in Table 5. This is consistent with the fact that the lowest significant difference in high versus low BGL PPG cycles were observed for the untreated type II diabetes subject S4. Least-squares regression analysis indicated a significant positive correlation between the predicted and reference glucose concentrations in all subjects (Table 6).

## 5. Discussion

The potential of the proposed method for continuous BGL monitoring is evidenced by 100% of the estimated BGL values falling within the clinically acceptable regions A and B of the CEG plots for all four subjects, as shown in Figure 10. The estimated CEG values in Region A fall within 20% of the reference (correct) BGL value, which has no negative effect on the clinical decisions, while the values in Region B of CEG are more than 20% apart from the reference BGL value, but this has low to no impact on clinical outcome [33] and does not lead to inappropriate treatment. This level of accuracy is particularly important for diagnosing hypoglycemia and hyperglycemia, where immediate intervention is required, as clinically wrong diagnosis may lead to the potentially fatal decision to inject insulin in patients with extremely low blood glucose levels. According to the tolerance range of the latest ISO standard (ISO: 15197:2015), the estimated blood glucose levels should be within ±15 mg/dL when the true value is <100 mg/dL, whereas values ≥100 mg/dL should be within ±20 mg/dL [34]. Observe from Figure 10 that the results for S1 and S2 meet these criteria with an average accuracy of 15 mg/dL (±9), while the estimated values for the type I diabetic S3 (accuracy: 19 ± 17 mg/dL) and type II diabetic S4 (35 ± 22 mg/dL) marginally fall outside the recommended range. This can be explained by the range of BGLs in this study being 2–3 times larger than those considered in the existing studies in the literature, with BGLs as high as 345.7 mg/dL for subject S4. For rigor, we also performed the analysis based on the median of PPG cycles, with the results very similar to those reported here which were obtained based on the means of PPG cycles.

Overall, the results obtained using the in-ear sensor are comparable to those obtained in previous studies for other PPG sites, as shown in Table 7. In [17] four different wavelengths were tested, with the best results obtained when all four wavelength measurements were used for estimating BGLs. The results in this work are consistent with the results in [17] when considering data within their tested glucose range (70–152 mg/dL) and the case where they use only two LEDs (880 nm and 940 nm) rather than four. While the choice of an optimal NIR wavelength for BGL monitoring is a subject of our continuing research, it is important to note that other glucose absorption peaks do exist at higher wavelengths [11,30,31], but some constituents of blood (e.g., water) also have absorption peaks at this range and would therefore mask the absorption by the glucose molecules.

In this work, the ear canal was used as a measurement site, owing to its unique and distinguishing advantages over body-worn sensing locations. These include:

1. Stable body/skin temperature, which in turn means that in-ear PPG measurements do not suffer from the adverse effects of vasoconstriction, the phenomenon of the constriction of blood vessels due to local body temperature changes, which in turn affects the amplitude of the PPG signal and thus may bias the estimated BG values [35];
2. Constant pressure/tension between the skin and the sensor yields stable and robust measurements (minimal day-to-day variation in the recordings);
3. The relative position between the head and the body remains mainly constant in most daily activities, which allows for truly continuous and stable long-term PPG measurements;
4. The in-ear sensor is generic and its viscoelastic nature means that it fits all ears;
5. Affordability and scalability, with only one off-the-shelf PPG sensor and only one wavelength needed for clinically acceptable accuracy.

## 6. Conclusions

This proof-of-concept study introduced an in-ear device for continuous blood glucose monitoring using an off-the-shelf pulse oximeter (PPG) sensor. An LED with a wavelength of 880 nm was used to acquire the PPG signal from which the blood glucose levels were estimated through machine learning. The subjects recruited for this study cover the whole spectrum of non-diabetic, pre-diabetic, and diabetic (type I and type II) conditions. The data were recorded over nine days while fasting and after the first daily meal (breakfast), over at least two hours. Statistically significant features were extracted from the PPG signals in order to capture the relationship between the PPG waveform and the reference glucose measurement from a commercially available glucometer. These features, when used for training ensemble regressors, resulted in the mean accuracy of 82% of the predicted BGLs residing within region A of the Clarke error grid (CEG). The highest clinical accuracy was achievable at around 90% of predicted values residing within region A of the CEG, while for all the subjects 100% of the predicted values reside within the CEG regions A and B—indicating no clinical misdiagnosis. Overall, this study demonstrated the potential of the ear canal as a site for non-invasive blood glucose monitoring. Our future work will focus on a large-scale study over a bigger cohort of subjects and longer monitoring hours (including between meals), which will facilitate the use of more advanced, data-hungry, machine learning techniques for BGL prediction from PPG.

## Figures and Tables

**Table 1:** Dataset description.

Subject	1	2	3	4
Gender	Female	Male	Male	Male
Age (years)	30	47	38	59
Glucose range (mg/dL)	75.6–142.2	86.4–194.4	63.0–174.6	183.6–345.7
No. of days between first and last recording	28	45	32	11
No. of finger pricks	56	60	44	84
No. of PPG recordings	56	60	44	84
No. of PPG cycles	6508	6435	6648	12476
Health status	Non-diabetic	Pre diabetic	Type I diabetic	Type II diabetic

**Table 2:** Summary of features extracted from each PPG cycle.

Feature	Description
DC value	Average value of the low-passed PPG signal
Peak value	Amplitude of the systolic peak [12]
Delta AC	Difference between the peak value and the trough on the left
Rise slope	Slope of the line between the trough on the left of the peak to the peak value
Rise time	Time difference between the trough on the left of the peak and the peak value
Fall level	Difference between the values of the peak and the trough on the right
Fall slope	Slope of the line between the trough on the right of the peak and the peak value
Fall time	Time between the trough on the right of the peak and the peak value
Width at 1/4 height	Value at 1/4 of the height of Delta AC
Width at 3/4 height	Value at 3/4 of the height of Delta AC
Base width	Difference between the trough on the right of the peak and the trough on its left
Area	Area enclosed between the two troughs
Optical density	$OD=\log(1+\frac{deltaAC}{DC})$ [17]
TKEO mean	Mean, variance, standard
TKEO variance	deviation, skewness and
TKEO std	kurtosis of the TKEO calculated
TKEO skewness	from each cycle using:
TKEO kurtosis	$x\left[n\right]=x^{2}\left[n\right]-x\left[n+1\right]x\left[n-1\right]$

**Table 3:** Description of the training and test data.

Subject	1	2	3	4
Total No. of cycles	6508	6435	6648	12,476
No. of valid cycles	5473	5419	4433	9935
No. of training cycles	3524	3859	3355	7607
No. of testing cycles	1949	1560	1078	2328
Training glucose range (mg/dL)	75.6–129.6	86.4–194.4	63.0–160.2	183.6–342
Testing glucose range (mg/dL)	88.2–142.2	95.4–153	75.6–174.6	199.8–345.7

**Table 4:** Optimal parameters of the ensemble regression models and the RMSE of the 5-fold cross-validation results.

Subject	1	2	3	4
Ensemble method (Bag/LSBoost)	Bag	Bag	LSBoost	LSBoost
No. of learners (10–500)	26	30	44	11
Minimum leaf size (1–18)	4	8	1	1
No. of predictors to sample (1–19)	1	1	19	19
RMSE (mg/dL)	11.98	27.93	20.59	39.03

**Table 5:** Predictions of the blood glucose levels.

Subject	% of Predictions in Region A of CEG	% of Predictions in Region A & B of CEG	RMSE (mg/dL)	Correlation (r) between the Predicted and Reference Value	Mean Absolute Relative Difference (MARD) (%)
S1	89.48	100	17.54	0.64	12.8
S2	83.33	100	17.78	0.54	13.3
S3	77.78	100	25.25	0.68	18.44
S4	77.77	100	42.78	0.56	13.5

**Table 6:** The regressed coefficients (slope and y-intercept), standard error (SE), and coefficient of determination ($R^{2}$).

Subject	Slope (95% C.I.)	y-Intercept (95% C.I.)	Standard Error (mg/dL)	$R^{2}$
S1	0.078 (0.029, 0.127)	94.05 (88.48, 99.62)	6.61	0.40
S2	0.351 (0.060, 0.641)	84.7 (47.99, 121.4)	47.54	0.29
S3	0.419 (0.010, 0.829)	70.59 (22.45, 118.7)	45.95	0.46
S4	0.255 (0.057, 0.454)	189.8 (135.1, 244.4)	78.28	0.32

**Table 7:** Related work.

Reference	Wavelength (nm)	CEG A (%)	Glucose Range (mg/dL)	Sensor Placement
Rachim et al. [17]	535, 660, 850, 950	100	70–152	Wrist
Ramasahayam et al. [29]	935, 950, 1070	95.38	70–180	Finger
Lee et al. [13]	940	88.57	75–230	Wrist
Hina et al. [16]	940	97.5	80–180	Finger
This work	880	82.05	63.0–345.7	Ear canal

## Data Availability

The data are not publicly available due to ethical restrictions.
